# Epidemiology of surgical occupational exposures in an infectious disease hospital: a 5-year retrospective study

**DOI:** 10.3389/fpubh.2026.1797229

**Published:** 2026-05-11

**Authors:** Peng Yan, Chunlin Cai, Xiaofang Liu, Liping Jiang, Yangzhen Liu

**Affiliations:** 1 Department of Interventional Vascular Surgery, Hunan Provincial People’s Hospital and the First-Affiliated Hospital of Hunan Normal University, Changsha, Hunan, China; 2 Department of Hospital Infection Management, The First Hospital of Changsha, Changsha, Hunan, China; 3 Hospital Office, The First Hospital of Changsha, Changsha, Hunan, China

**Keywords:** epidemiological investigation, infectious disease hospital, occupational exposure, sharp injuries, surgery

## Abstract

**Objective:**

To analyze the epidemiological characteristics and associated factors at the univariate level of surgical occupational exposures in an infectious disease hospital over the past 5 years, and to provide evidence for developing targeted prevention strategies.

**Methods:**

We retrospectively collected surgical occupational exposure reports from an infectious disease hospital between January 2020 and December 2024, including 104 valid cases. Statistical analysis was performed using R 4.4.2 software to describe the temporal, demographic, departmental, role-based, and exposure-type distributions, along with a univariate analysis.

**Results:**

The incidence of occupational exposure showed an increasing trend year by year, with a significant rise in 2024. Males accounted for 71.2% of the exposures, with the majority aged 25–39 years. Surgical assistants had the highest exposure proportion (50.0%), and the departments with the most exposure were Infectious Surgery and Orthopedics (18.3% each). The primary type of exposure was sharp injuries (60.6%). Significant differences were observed among the different surgical roles in terms of sex, age, education, and department (*p* < 0.001). Emergency surgery exposure was concentrated in Orthopedics (46.2%) and Obstetrics (15.4%), predominantly involving grade 2–3 surgeries.

**Conclusion:**

This study revealed a heterogeneous risk pattern for surgical occupational exposure in an infectious disease hospital. Surgical assistants and the Orthopedics/Infectious Disease Surgery departments constituted the core high-risk cluster, confirming that job role and departmental specialty are more critical determinants of exposure risk than individual experience. Notably, the recurrence of nearly half of all exposure events provides compelling evidence that current prevention strategies—centered predominantly on training and personal protective equipment—exhibit structural failure in effectively breaking the chain of exposure. Therefore, effective risk control must transcend individual behavioral discipline and shift toward a precision prevention system anchored in engineering controls and systemic workflow interventions.

## Introduction

Surgical occupational exposure in infectious disease hospitals poses a significant threat to healthcare workers, potentially leading to professional infections, psychological distress, and public health concerns. Existing studies indicate that such exposures are frequent among surgical staff, particularly in departments such as General Surgery and Obstetrics & Gynecology, with blood or body fluid exposure being the most common type (1).

Multiple factors contribute to occupational exposure, particularly the utilization of personal protective equipment and compliance with infection prevention and control practices (2). After an exposure event, healthcare workers are susceptible to anxiety and other negative emotions, often reflected in significantly worse mental health scores. This reality highlights the need for effective pre-job education and standardized management protocols (3). Moreover, repeated exposure is strongly linked to the type of personnel, surgical site, and timing of the operation, indicating that ongoing monitoring is essential for addressing occupational exposure (4).

From an occupational health and safety perspective, surgical occupational exposure stems largely from systemic deficiencies rather than isolated individual errors. The literature indicates that traditional exposure models in infectious disease hospitals often “neglect the nuances of experience-dependent risk, leading to persistent prevention gaps” (5), underscoring the inadequate recognition of systemic risk factors in current strategies. Multiple studies emphasize the limitations of prevention models that rely excessively on training and personal protective equipment (PPE), noting that effective management of high-consequence infectious diseases requires a “systems-based strategy” focused on early detection, timely initiation of infection prevention and control measures, and appropriate PPE use, rather than isolated reinforcement of protective equipment (6).

The value of a human factors engineering perspective in risk management is increasingly being recognized. One study, by establishing a risk knowledge platform integrating “human factors engineering, data science, and data governance,” systematically managed healthcare-associated infection risks in an acute hospital setting; the 2-year implementation validated the positive impact of multidisciplinary collaboration on workflow optimization and exposure risk reduction (7). Research among frontline nurses further confirms that “repeated occupational exposure and elevated stress and fatigue levels” significantly increase infection risk (e.g., COVID-19) and reveals the intrinsic associations among work resources, team collaboration, work-life balance, and burnout (8), thereby corroborating the amplifying effect of systemic factors such as fatigue and time pressure on exposure risk.

Nevertheless, the literature reveals a gap between practice and the ideal framework. Although agencies including the WHO, CDC, and OSHA advocate for systemic prevention strategies (9–11), empirical research specifically focused on the surgical environment within infectious disease hospitals remains scarce. To address this gap, this study analyzed surgical occupational exposure reports submitted by healthcare workers at a tertiary infectious disease hospital between 2020 and 2024. We systematically examined the incidence and influencing factors to optimize protective strategies and reduce infection risks among medical staff. This cohort study was reported in accordance with the STROCSS guidelines.

## Materials and methods

### Study setting

This single-center retrospective study was conducted at The First Hospital of Changsha, a provincial-level designated tertiary hospital (Grade 3 Class A) specializing in infectious disease prevention and treatment in Hunan Province, China. The hospital has 2,000 beds and 15 surgical departments. Approximately 480 healthcare workers regularly engage in surgical operations, including attending surgeons, resident physicians, anesthesiologists, and operating room nurses. Between 2020 and 2024, the annual surgical volume ranged from 9,797 to 14,958.

### Variable definitions

The following variables were extracted and categorized as follows:

Emergency surgery: A surgical procedure performed within 24 h of admission for an urgent or life-threatening condition (e.g., trauma, acute abdomen, obstetric emergency).

Grade of surgery: Classified according to the Chinese surgical grading system (grades 1–4), with higher grades indicating greater complexity and potential risk.

Surgical role: Categorized as primary surgeon (lead operating surgeon), surgical assistant (first or second assistant), anesthesiologist, or others (including scrub nurses and circulating nurses).

Time of exposure: Normal working hours were defined as 08:00–12:00 and 14:30–18:00, Monday through Friday. Unsocial working hours include nights, weekends, public holidays, and all other times outside the defined normal working hours.

Exposure type: Needlestick or sharp injury (injury caused by needles, scalpels, or other sharp instruments) vs. contact exposure (splash of blood or body fluids onto mucous membranes or non-intact skin).

Exposure level: Classified into three levels based on the route and extent of exposure. Level 1 is defined as the exposure of damaged skin or mucous membranes to a source with a small volume and short duration. Level 2 includes either (a) exposure of damaged skin or mucous membranes to a source with a large volume and prolonged duration or (b) percutaneous injury with mild damage (e.g., superficial abrasion or needlestick). Level 3 refers to percutaneous injury with severe damage (e.g., deep wound or visibly blood-contaminated sharp instrument).

Prior safety training: Completion of any hospital-organized occupational safety or infection control training session within 12 months preceding the exposure event.

### History of prior exposure: self-reported previous bloodborne occupational exposure events occurring during the individual’s employment at this hospital

#### Study subjects

This study used a retrospective cohort design. All occupational exposure incidents formally reported through the hospital.

Data were extracted from the electronic occupational exposure report database (2020–2024) via the hospital-acquired infection surveillance system. To ensure accuracy, these data were cross-verified with multiple sources, including the Healthcare Worker Occupational Exposure Registration Form from the hospital’s OA workflow system and linked patient data and surgical records from the electronic medical record system. The collected data encompassed three main categories: (1) Exposed Individual Information: gender, age, education level, job position, years of service, and department; (2) Exposure Characteristics: time of occurrence, surgical role, surgery grade, emergency status, exposure type, exposure level, and severity; and (3) Management Factors: history of safety training, previous exposure history, time to reporting, and completeness of post-exposure management. A questionnaire file was created using EpiData 3.1 software, and logical validation rules were implemented to prevent entry errors. All data were independently entered by two trained researchers to ensure their accuracy. The study protocol was reviewed and approved by the Ethics Committee of the Tertiary Infectious Disease Hospital. The requirement for informed consent was waived because of the retrospective nature of the study and the use of anonymized data.

#### Statistical analysis

Statistical analysis was performed using R software (version 4.4.2). Categorical data were described using frequency and proportion. The chi-square test was used for between-group comparisons of nominal categorical variables [e.g., sex, department, surgical role (Tables 1, 2), and exposure type (Table 3)]. The Pearson’s Chi-squared test was applied to ordinal categorical variables (e.g., surgery grade and exposure level). Variables were selected for analysis based on clinical relevance and prior literature on occupational exposure risk factors. Multivariable adjustment was not performed due to the limited sample size (*n* = 104) and the study’s exploratory, descriptive nature. All tests were two-sided, and statistical significance was set at *p* < 0.05.

**Table 1:** Occupational exposure risk analysis of different surgical roles.

Variable	Overall *N* = 104^1^	Primary surgeon *N* = 27^1^	Surgical assistant *N* = 52^1^	Anesthesiologist *N* = 8^1^	Other *N* = 17^1^	Statistic^2^	p-value^2^
Sex						24.8	**<0.001**
Male	74 (71%)	23	43	3	5		
Female	30 (29%)	4	9	5	12		
Age (years)						70.3	**<0.001**
20–24	8 (7.7%)	0	3	0	5		
25–29	26 (25%)	0	22	1	3		
30–34	11 (11%)	0	8	0	3		
35–39	26 (25%)	6	11	4	5		
40–44	17 (16%)	8	6	3	0		
45–49	13 (13%)	10	2	0	1		
50–54	3 (2.9%)	3	0	0	0		
Education						43.3	**<0.001**
Associate degree	2 (1.9%)	0	0	0	2		
Bachelor’s degree	30 (29%)	7	8	1	14		
Master’s degree	72 (69%)	20	44	7	1		
Position						156	**<0.001**
Resident training physician	4 (3.8%)	0	4	0	0		
Resident physician	28 (27%)	0	27	1	0		
Attending physician	20 (19%)	2	14	3	1		
Associate chief physician	28 (27%)	16	7	4	1		
Chief physician	9 (8.7%)	9	0	0	0		
Nurse	11 (11%)	0	0	0	11		
Nurse-in-charge	4 (3.8%)	0	0	0	4		
Years of service						46.6	**<0.001**
≦2 years	22 (21%)	0	17	1	4		
2–5 years	22 (21%)	0	18	0	4		
5–10 years	13 (13%)	2	8	2	1		
>10 years	47 (45%)	25	9	5	8		
Department						210	**<0.001**
Obstetrics	2 (1.9%)	1	1	0	0		
Otorhinolaryngology	7 (6.7%)	2	5	0	0		
Gynecology	2 (1.9%)	0	2	0	0		
Infectious disease surgery	19 (18%)	4	14	0	1		
Orthopedics	19 (18%)	11	8	0	0		
Spinal surgery	4 (3.8%)	0	3	0	1		
Anesthesiology	9 (8.7%)	0	0	8	1		
Urology	3 (2.9%)	3	0	0	0		
Gastrointestinal surgery	5 (4.8%)	2	3	0	0		
Breast and thyroid surgery	7 (6.7%)	2	5	0	0		
Hepatobiliary surgery	5 (4.8%)	0	5	0	0		
Nephrology and rheumatology	2 (1.9%)	2	0	0	0		
Operating room	12 (12%)	0	0	0	12		
Cardiology	1 (1.0%)	0	0	0	1		
Cardiothoracic surgery	6 (5.8%)	0	6	0	0		
Ophthalmology	1 (1.0%)	0	0	0	1		

1 *n* (%).

2 Pearson’s Chi-squared test. Bold values indicate statistical significance level is *p* < 0.05.

**Table 2:** Risk analysis of occupational exposure in emergency surgery.

Variable	Overall *N* = 104^1^	Emergency *N* = 13^1^	Non-emergency surgeries *N* = 91^1^	Statistic^2^	p-value^2^
Department				27.3	**0.026**
Obstetrics	2 (1.9%)	2	0		
Otorhinolaryngology	7 (6.7%)	0	7		
Gynecology	2 (1.9%)	0	2		
Infectious disease surgery	19 (18%)	2	17		
Orthopedics	19 (18%)	6	13		
Spinal surgery	4 (3.8%)	0	4		
Anesthesiology	9 (8.7%)	1	8		
Urology	3 (2.9%)	0	3		
Gastrointestinal surgery	5 (4.8%)	1	4		
Breast and thyroid surgery	7 (6.7%)	0	7		
Hepatobiliary surgery	5 (4.8%)	1	4		
Nephrology and rheumatology	2 (1.9%)	0	2		
Operating room	12 (12%)	0	12		
Cardiology	1 (1.0%)	0	1		
Cardiothoracic surgery	6 (5.8%)	0	6		
Ophthalmology	1 (1.0%)	0	1		
Grade of surgery				13.4	**0.004**
Grade 1	17 (16%)	2	15		
Grade 2	11 (11%)	5	6		
Grade 3	45 (43%)	5	40		
Grade 4	31 (30%)	1	30		

1 *n* (%).

2 Pearson’s Chi-squared test. Bold values indicate statistical significance level is *p* < 0.05.

**Table 3:** Risk analysis of different types of occupational exposure.

Variable	Overall *N* = 104^1^	Needlestick or sharps injury *N* = 63^1^	Contact exposure *N* = 41^1^	Statistic^2^	p-value^2^
Time of exposure				7.01	**0.008**
Normal working hours	56 (54%)	41	15		
Unsocial working hours	48 (46%)	22	26		

1 *n* (%).

2 Pearson’s Chi-squared test. Bold values indicate statistical significance level is *p* < 0.05.

## Results

### Temporal distribution characteristics of occupational exposures

Between 2020 and 2024, the designated infectious disease hospital exhibited a year-on-year increase in the number of surgery-related occupational exposures. Throughout this period, the total number of surgical procedures demonstrated gradual growth, paralleled by a rise in the annual exposure incident count and annual incidence rate. Notably, the annual incidence rate showed the most pronounced increase in 2024 (Table 4).

**Table 4:** Annual statistics of operation-related occupational exposure events.

Variable	2020	2021	2022	2023	2024
Number of surgeries with occupational exposures	8	18	18	24	36
Total number of surgeries	9,797	13,102	13,758	14,944	14,958
Annual incidence rate (%)	0.082	0.137	0.131	0.161	0.241

**Figure 1:**
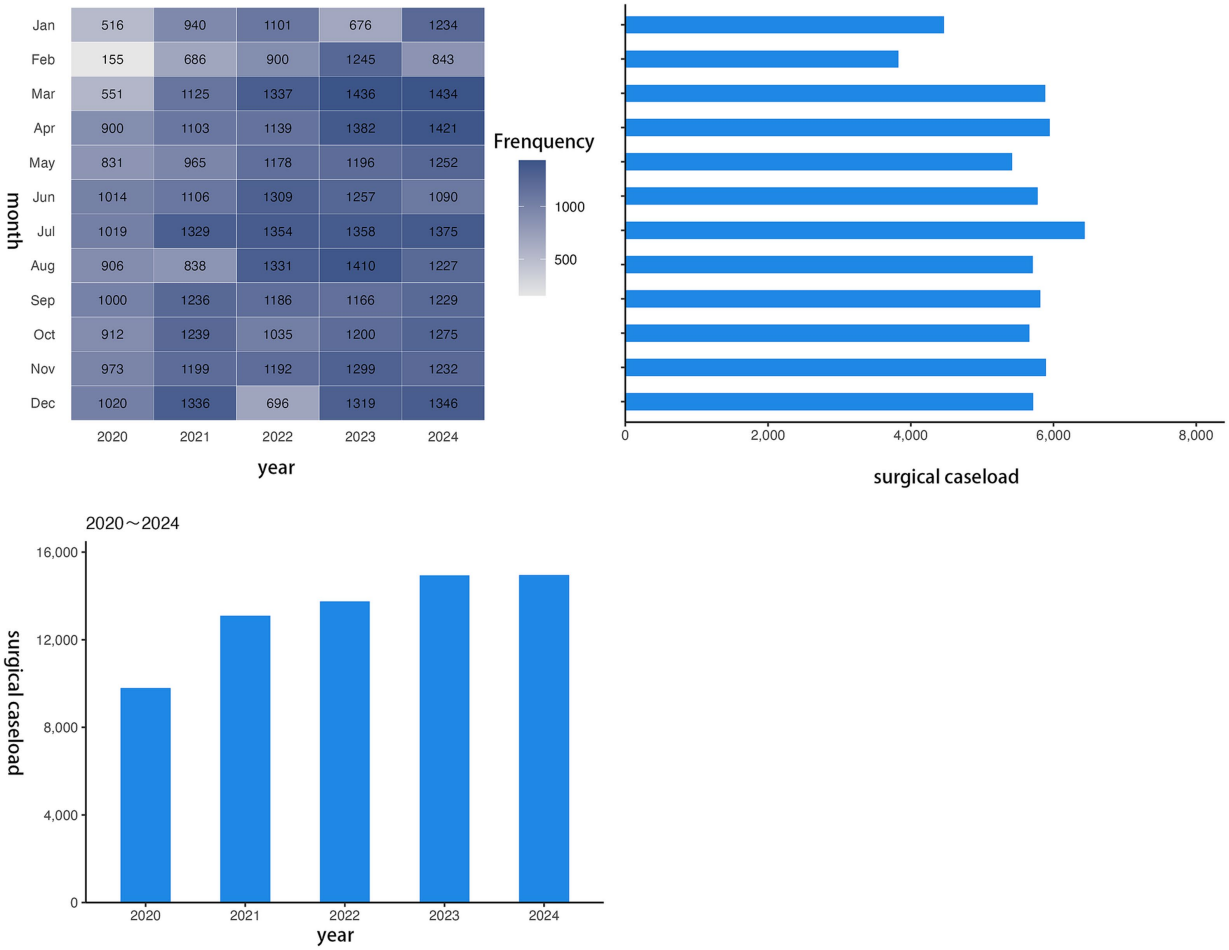
Time distribution of the number of operations from 2020 to 2024.

**Figure 2:**
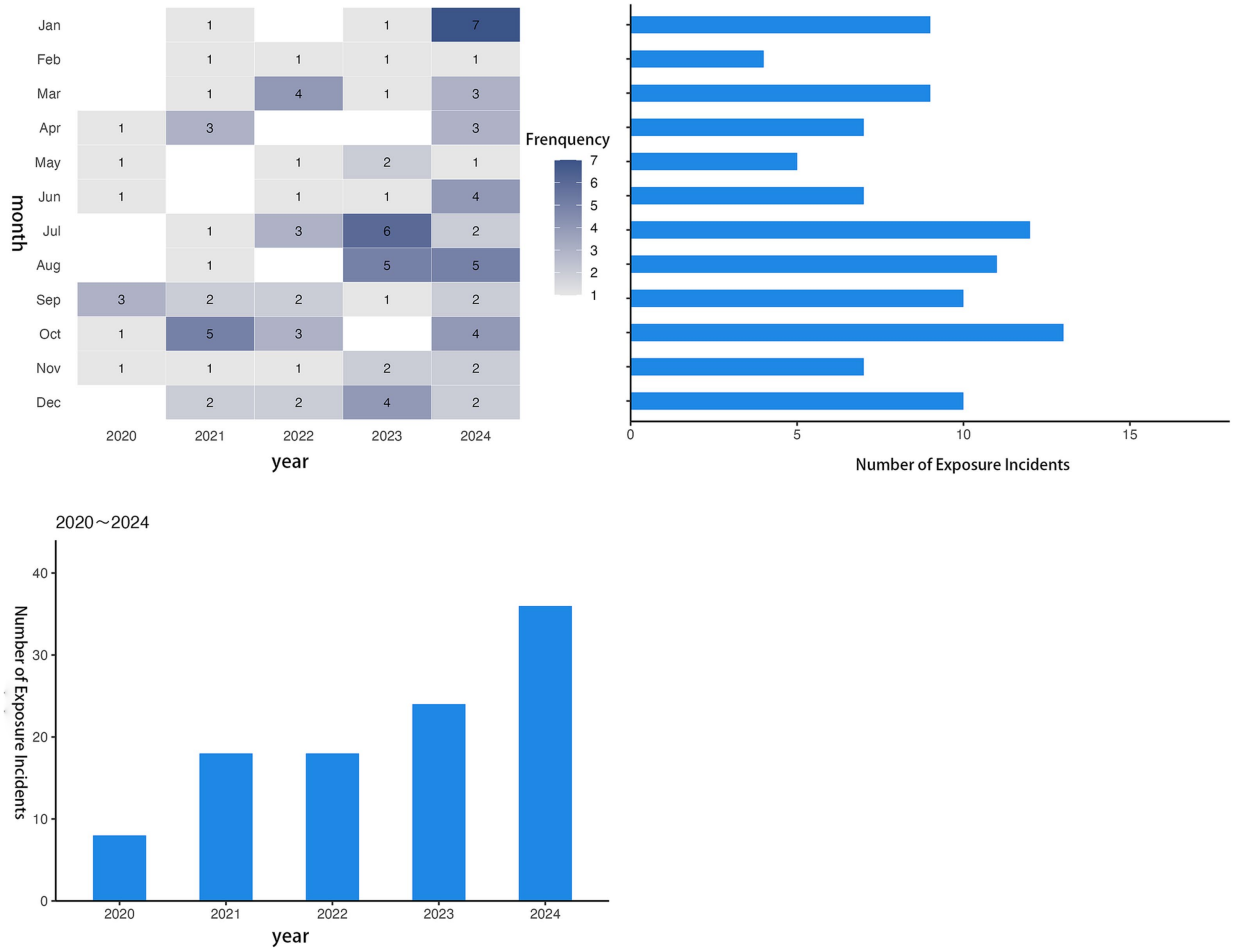
Time distribution of operation-related occupational exposures.

Analysis of the monthly distributions of surgical caseloads and associated occupational exposures over the past 5 years indicated a correlative pattern at the study hospital. The monthly variation in surgical volume exhibited a consistent annual trend with distinct seasonal fluctuations (Figure 1). Specifically, the caseload was relatively low during the first quarter (January to March), with a particularly notable decrease in January and February, and the time distribution of surgery-related occupational exposures largely corresponded to the fluctuation trend in surgical volume. Months with higher surgical caseloads generally experienced a higher frequency of exposure events. Moreover, the monthly number of exposure events from July to December was consistently greater than that from January to June during the study period (Figure 2).

### Characteristics of the exposed population and exposure events

A total of 104 cases of surgery-related occupational exposure were included in this study. Of these, 74 cases (71.2%) involved males and 30 cases (28.8%) involved females. The age distribution was primarily concentrated in the 25–29 (26 cases, 25.0%) and 35–39 (26 cases, 25.0%) year groups, with the 50–54 years group accounting for the lowest proportion (3 cases, 2.9%). Regarding education, individuals holding a master’s degree constituted the largest group (72 cases, 69.2%), while only 2 cases (1.9%) involved individuals with an associate degree. In terms of professional roles, associate chief physicians (28 cases, 26.9%), resident physicians (28 cases, 26.9%), and attending physicians (20 cases, 19.2%) were the main groups affected. Nurses and nurse-in-charges accounted for 15 cases (14.4%). Based on years of experience, individuals with > 10 years of service represented the highest proportion (47 cases, 45.2%), followed by 22 cases each (21.2%) in the ≤2 years and 2–5 years groups. The most frequently involved departments were Infectious Disease Surgery and Orthopedics (19 cases each, 18.3%), followed by the Operating Room (12 cases, 11.5%) and Anesthesiology Department (9 cases, 8.7%). Regarding surgical roles, surgical assistants accounted for the highest proportion (52 cases, 50.0%), followed by primary surgeons (27 cases, 26.0%), anesthesiologists (8 cases, 7.7%), and other personnel (17 cases, 16.3%). Concerning the timing of exposure, 56 cases (53.8%) occurred during normal working hours, whereas 48 cases (46.2%) occurred outside normal working hours. In terms of reporting time, 53 cases (51.0%) were reported within 1 h, and 41 cases (39.4%) were reported 2 h or later after the incident. Fifty-four individuals (51.9%) had received prior occupational safety training, and 51 (49.0%) reported a history of occupational exposure. The predominant exposure type was needlestick or sharp injury (63 cases, 60.6%), followed by contact exposure (41 cases, 39.4%). In terms of surgical grade, Grade 3 surgeries were most common (45 cases, 43.3%), followed by Grade 4 surgeries (31 cases, 29.8%) and emergency surgeries (13 cases, 12.5%). The exposure level was primarily Level 2 (53 cases, 51.0%), followed by Level 3 (28 cases, 26.9%). Regarding postexposure management, complete follow-up testing and prophylactic medication were administered in 69 cases (66.3%), whereas 10 cases (9.6%) received no intervention (Table 5).

**Table 5:** Basic situation of occupational exposure population related to operation.

Variable	Overall *N* = 104^1^	Male *N* = 74^1^	Female *N* = 30^1^	Statistic^2^	*p*-value^2^
Age (years)				7.22	0.30
20–24	8 (7.7%)	5	3		
25–29	26 (25%)	19	7		
30–34	11 (11%)	7	4		
35–39	26 (25%)	15	11		
40–44	17 (16%)	13	4		
45–49	13 (13%)	12	1		
50–54	3 (2.9%)	3	0		
Education				8.29	**0.016**
Associate’s degree	2 (1.9%)	0	2		
Bachelor’s degree	30 (29%)	18	12		
Master’s degree	72 (69%)	56	16		
Position				25.6	**<0.001**
Resident training physician	4 (3.8%)	3	1		
Resident physician	28 (27%)	24	4		
Attending physician	20 (19%)	13	7		
Associate chief physician	28 (27%)	24	4		
Chief physician	9 (8.7%)	7	2		
Nurse	11 (11%)	2	9		
Nurse-in-charge	4 (3.8%)	1	3		
Years of service				2.36	0.50
≦2 years	22 (21%)	16	6		
2–5 years	22 (21%)	17	5		
5–10 years	13 (13%)	7	6		
>10 years	47 (45%)	34	13		
Department				53.3	**<0.001**
Obstetrics	2 (1.9%)	0	2		
Otorhinolaryngology	7 (6.7%)	4	3		
Gynecology	2 (1.9%)	0	2		
Infectious disease surgery	19 (18%)	18	1		
Orthopedics	19 (18%)	18	1		
Spinal surgery	4 (3.8%)	4	0		
Anesthesiology	9 (8.7%)	3	6		
Urology	3 (2.9%)	3	0		
Gastrointestinal surgery	5 (4.8%)	5	0		
Breast and thyroid surgery	7 (6.7%)	4	3		
Hepatobiliary surgery	5 (4.8%)	5	0		
Nephrology and rheumatology	2 (1.9%)	2	0		
Operating room	12 (12%)	3	9		
Cardiology	1 (1.0%)	0	1		
Cardiothoracic surgery	6 (5.8%)	5	1		
Ophthalmology	1 (1.0%)	0	1		
Surgical role				24.8	**<0.001**
Primary surgeon	27 (26%)	23	4		
Surgical assistant	52 (50%)	43	9		
Anesthesiologist	8 (7.7%)	3	5		
Others	17 (16%)	5	12		
Time of exposure				0.081	0.78
Normal working hours	56 (54%)	41	15		
Unsocial working hours	48 (46%)	33	15		
Time to reporting				0.672	0.71
<1 h	53 (51%)	36	17		
1–2 h	10 (9.6%)	7	3		
≧2 h	41 (39%)	31	10		
Prior safety training				0.000	>0.99
Yes	54 (52%)	38	16		
No	50 (48%)	36	14		
History of prior exposure				3.32	0.068
Yes	51 (49%)	41	10		
No	53 (51%)	33	20		
Type of exposure				1.40	0.24
Needlestick or sharps injury	63 (61%)	48	15		
Contact exposure	41 (39%)	26	15		
Grade of surgery				4.44	0.22
Grade 1	17 (16%)	12	5		
Grade 2	11 (11%)	10	1		
Grade 3	45 (43%)	28	17		
Grade 4	31 (30%)	24	7		
Emergency surgery				0.027	0.87
Yes	13 (13%)	10	3		
No	91 (88%)	64	27		
Exposure level				5.73	0.057
Level 1	23 (22%)	12	11		
Level 2	53 (51%)	42	11		
Level 3	28 (27%)	20	8		
Post-exposure management				0.886	0.64
Completed all follow-up and prophylaxis	69 (66%)	51	18		
Partially completed	25 (24%)	16	9		
Not performed	10 (9.6%)	7	3		
Years				4.33	0.36
2020	8 (7.7%)	5	3		
2021	18 (17%)	12	6		
2022	18 (17%)	16	2		
2023	24 (23%)	18	6		
2024	36 (35%)	23	13		

1 *n* (%).

2 Pearson’s Chi-squared test. Bold values indicate statistical significance level is *p* < 0.05.

### Occupational exposure risk characteristics by surgical role

The univariate analysis of occupational exposure risk across different surgical roles revealed statistically significant differences in sex, age, education, position, years of service, and department among the 104 participants (all *p* < 0.001). Regarding sex distribution, males constituted a higher proportion among primary surgeons (85.2%) and surgical assistants (82.7%). In contrast, females were more prominent among anesthesiologists (62.5%) and “other” roles (70.6%). In terms of age distribution, primary surgeons were predominantly aged 40 years and above (40–44 years: 29.6%; 45–49 years: 37.0%). Surgical assistants were primarily aged 25–34 years (25–29 years: 42.3%; 30–34 years: 15.4%). Most anesthesiologists were aged 35–44 years (35–39 years: 50.0%; 40–44 years: 37.5%). The “other” role group was mainly composed of individuals aged 20–24 years and 35–39 years (29.4% each group). Regarding education level, individuals with a master’s degree exceeded 70% among primary surgeons (74.1%), surgical assistants (84.6%), and anesthesiologists (87.5%). The “other” roles were predominantly bachelor’s degree holders (82.4%), with an additional 11.8% holding an associate’s degree. Position distribution showed that primary surgeons were mainly associate chief physicians (59.3%) and chief physicians (33.3%). The surgical assistants were primarily resident physicians (51.9%) and attending physicians (26.9%). Anesthesiologists were concentrated among attending physicians (37.5%) and associate chief physicians (50.0%). The “other” roles consisted mainly of nurses (64.7%) and nurses-in-charge (23.5%). Regarding years of service, 92.6% of primary surgeons had more than 10 years of experience. Surgical assistants mostly had ≤5 years of service (≤2 years: 32.7%; 2–5 years: 34.6%). Most anesthesiologists had >10 years of experience (62.5%). Among the “other” roles, the proportions of those with >10 years and ≤5 years of service were similar (47.1% vs. 47.0%). Departmental distribution indicated that primary surgeons were mainly from Orthopedics (40.7%) and Infectious Disease Surgery (14.8%) departments. Surgical assistants were primarily from the Department of Infectious Disease Surgery (26.9%) and Orthopedics (15.4%). All anesthesiologists (100.0%) were from the Department of Anesthesiology. The “other” roles were predominantly from the Operating Room (70.6%).

### Risk characteristics of occupational exposures in emergency surgeries

The univariate analysis of occupational exposure risk in emergency surgeries revealed that among the 104 surgery-related exposure cases, statistically significant differences were observed between emergency and non-emergency surgeries in terms of department, time of exposure, and surgery grade (*p* < 0.05). Regarding departmental distribution, Orthopedics accounted for the highest proportion of exposure during emergency surgeries (46.2%, 6/13), followed by Obstetrics (15.4%, 2/13). In contrast, the proportional differences between emergency and non-emergency exposures were relatively smaller in departments such as Infectious Disease Surgery and Anesthesiology. Exposure during non-emergency surgeries was distributed across multiple departments, including Infectious Disease Surgery (18.7%) and Orthopedics (14.3%). In terms of surgery grade, grade 2 (38.5%, 5/13) and grade 3 (38.5%, 5/13) surgeries were prominent among emergency surgery exposures. For non-emergency surgeries, exposure occurred predominantly during grade 3 (44.0%, 40/91) and grade 4 (33.0%, 30/91) procedures.

### Risk characteristics by type of occupational exposure

Among the 104 occupational exposure incidents, there were 63 cases (60.6%) of needlestick or sharp injuries and 41 cases (39.4%) of contact exposure. A significant difference was observed in the temporal distribution between the two exposure types (*p* < 0.05). Specifically, needlestick or sharp injuries occurred more frequently during normal working hours (65.1%, 41/63), whereas contact exposures were predominantly concentrated during unsocial working hours (63.4%, 26/41).

## Discussion

The risk of occupational exposure to infectious materials is inherently higher in designated infectious disease hospitals. This study found that the annual incidence of surgery-related occupational exposure at the study hospital has shown an increasing trend over the past 5 years. Notably, this trend did not fully parallel the changes in the total surgical caseload. Although the total number of surgeries in 2024 remained similar to that in 2023, the incidence of occupational exposure increased significantly. This suggests that the increasing risk of occupational exposure is not solely driven by an increase in surgical volume, and other contributing factors may be involved. Research indicates that an increase in surgical volume often leads to extended working hours for healthcare staff, which may serve as an indirect explanation for the association between surgical volume and occupational exposure risk (1). Concurrently, factors such as noncompliance with personal protective behaviors, gaps in organizational management, inadequate training, and the high-risk nature of specific departments or positions are also closely linked to the occurrence of occupational exposures (1, 12, 13). Further analysis of the monthly distribution patterns revealed an overall correlation between the surgical caseload and the number of exposure incidents. However, the probability of occupational exposure was higher in the second half of the year. This may be attributable to the relatively high surgical volume during this period, leading to cumulative fatigue from prolonged high workloads among staff and a subsequent decline in compliance with standardized protective operations (12). Seasonal factors may also play a role; for instance, the seasonal characteristics of certain infectious diseases could increase the complexity of patients’ conditions and elevate procedural risks during surgery (14). Future studies should delve deeper into the factors influencing occupational exposure. By integrating the patterns of surgical volume fluctuations, healthcare workers’ operational status, and disease characteristics, targeted prevention and control strategies can be formulated to reduce the risk of occupational exposure.

The demographic characteristics of surgery-related occupational exposures revealed a distinct sex disparity, with males accounting for 71.2% of the cases. This likely reflects the higher proportion of male physicians in surgical teams (15). The age distribution indicated that young and middle-aged physicians between 25 and 39 years old constituted the primary exposed group, a finding consistent with international studies, which may be attributed to their high level of surgical involvement coupled with relatively less experience (1). Notably, individuals with higher education levels and senior physicians were prominently represented among exposure cases, suggesting that even well-trained and experienced medical personnel remain at significant risk. In terms of professional roles, associate chief physicians and resident physicians exhibited the highest exposure rates. Combined with the finding that surgical assistants accounted for over half of all exposures by surgical role, this highlights the elevated risk associated with intraoperative assistance procedures. Multiple studies have identified suture needles as a leading cause of sharps injuries in the operating room (16–18). Departmental analysis showed that Infectious Disease Surgery and Orthopedics ranked equally highest in exposure frequency, possibly due to the greater use of sharp instruments and contact with exposure sources in these specialties. Previous research has indicated that 80–90% of orthopedic surgeons experience needlestick injuries (18). Sharp injuries were the predominant exposure type, consistent with the global epidemiological profile of healthcare occupational exposures (19, 20). Notably, nearly half (49%) of the exposed individuals had a prior history of occupational exposure, and 51.9% had received occupational safety training prior to the incident, suggesting persistent gaps in the implementation of current protective systems or a disconnect between training content and actual clinical risks. Relying solely on individual-level knowledge dissemination and behavioral training cannot effectively counteract deep-rooted systemic risks, such as excessive workload, irrational shift scheduling, inadequate staffing, and a weak safety culture (21–23). Studies indicate that occupational exposure risk factors span multiple dimensions, including organizational management, physical environment, and psychosocial aspects, and require systematic assessment using tools such as job exposure matrices to identify and intervene in structural problems (22). Without workflow optimization, establishment of supportive supervisory mechanisms, and cultivation of a robust safety culture, training alone is insufficient to interrupt the exposure chain (21, 23). Delays in reporting and instances of inadequate post-exposure management also reveal shortcomings in healthcare workers’ awareness of reporting protocols and the hospital’s subsequent follow-up and monitoring systems. Research by Yun et al. noted that medical students and surgeons are high-risk groups for sharps injuries but often underreport them. They recommended specialized skill training, destigmatizing reporting, and optimizing reporting procedures to enhance workplace safety (24). Moving forward, training content and methods should be tailored to different positions and years of experience, with a particular focus on high-risk areas, such as surgical assistants and intraoperative assisting procedures. The processes for handling sharps should be refined, and reporting and follow-up management must be strengthened to reduce the risk of occupational exposure and its adverse consequences.

This study found significant differences in the risk of occupational exposure among different roles within the surgical team. These differences were closely related to the specific responsibilities and operational characteristics of each position in the study. Primary surgeons were predominantly male, highly experienced, and highly educated senior professionals, frequently concentrated in high-risk departments such as Orthopedics and Infectious Disease Surgery. Their occupational exposure risk may stem from the inherent complexity of surgical procedures (25) and potential neglect of protective protocols when managing urgent intraoperative situations (26). Surgical assistants were primarily young, less experienced resident, and attending physicians, with a high proportion of males. Frequent assistance with tasks such as instrument passing and tissue suturing significantly increases the risk of sharps injuries (18). The anesthesiologist group was characterized by middle age, high education level, and substantial experience. Exposure hazards, such as needlesticks and blood splashes, exist during airway management and puncture procedures (20). The “Other” roles consisted mainly of female nurses concentrated in the operating rooms. Serving as circulating or scrub nurses, they were responsible for instrument preparation, intraoperative supply, and postoperative instrument transportation and cleaning, leading to frequent contact with contaminated equipment. Coupled with a high proportion of junior staff potentially lacking full risk awareness and protective proficiency, their risk profile was distinct. These role-based disparities in risk underscore the need to develop differentiated protection strategies tailored to the specific characteristics of each position, thereby enhancing the precision and effectiveness of preventive measures.

Significant differences in risk factors related to occupational exposure between emergency and non-emergency surgeries reveal distinct patterns of exposure risk for these two categories. Orthopedics accounted for a markedly higher proportion of exposure in emergency surgeries than in non-emergency procedures. This may be attributed to the fact that orthopedic emergencies often involve traumatic cases in which healthcare workers frequently use sharp instruments during wound management and fracture reduction. Coupled with substantial bleeding from patient wounds, this significantly increases the risk of blood exposure in healthcare workers. In obstetric emergency surgeries, exposure to amniotic fluid, blood, and other body fluids during delivery, combined with the time pressure of urgent cesarean sections, which may compromise the full implementation of protective measures, contributes to an elevated risk of occupational exposure. Emergency surgery exposures were primarily associated with grade 2 and 3 procedures, whereas exposures in non-emergency surgeries were concentrated in grade 3 and 4 operations. This suggests that both the urgency and complexity of surgery may be related to occupational exposure risk; therefore, for the prevention and control of occupational exposure in emergency surgeries, focused attention should be given to departments such as Orthopedics and Obstetrics. Concurrently, optimizing emergency surgical workflows and strengthening preoperative protective measures are essential to reduce the risk of occupational exposure.

The significant difference in temporal distribution between needlestick/sharp injuries and contact exposure is consistent with previous descriptions of the temporal characteristics of occupational exposures. Studies indicate that sharps injuries represent the most prominent occupational hazard in healthcare settings (27), and their occurrence patterns are closely linked to workflow. In this study, 65.1% of sharps injuries occurred during normal working hours, likely due to concentrated surgical volumes and increased procedural frequency leading to cumulative risk (18), highlighting that instrument handling and disposal during routine surgical procedures remain key focal points for protection. Conversely, the concentration of contact exposures during unsocial working hours (63.4%) may be associated with high time pressure and non-standardized use of personal protective equipment in emergency situations (28). This divergence in temporal distribution suggests the need for time-specific prevention strategies tailored to different exposure types. During non-emergency periods, emphasis should be placed on strengthening safety protocols for sharps management, whereas during emergency periods, priority should be given to monitoring and ensuring the standardized use of protective equipment.

In summary, this study revealed the epidemiological characteristics of surgery-related occupational exposures in a designated infectious disease hospital through multidimensional analysis, providing important evidence for formulating targeted prevention and control strategies. However, as the study is based on single-center data, the findings may not be fully representative of the occupational exposure landscape in infectious disease hospitals in different regions and scales.

Furthermore, this study revealed a heterogeneous risk pattern for surgical occupational exposure in an infectious disease hospital. Surgical assistants and the Orthopedics and Infectious Disease Surgery departments constituted the core high-risk cluster, confirming that job role and departmental specialty are more critical determinants of exposure risk than individual experience. The pronounced concentration of contact exposures in emergency orthopedic surgeries and the persistently high incidence of sharps injuries during high-complexity nonemergency procedures further corroborate the spatiotemporal heterogeneity of risk. Most importantly, nearly half of all exposure events were recurrent, and over half of the exposed individuals had received prior safety training—compelling evidence that current prevention strategies centered on training and personal protective equipment exhibit structural failure. Consequently, effective risk control necessitates a paradigm shift from reliance on individual behavioral discipline to a precision prevention system anchored in engineering controls and systemic workflow interventions. Specifically, hands-free instrument passing techniques should be mandated for surgical assistants; blunt-tip suture needles should be promoted for high-risk procedures, accompanied by contextualized protective prompts embedded in preoperative time-out checklists; and exposure events should be incorporated into root cause analysis frameworks for workflow review and task redesign, thereby constructing a resilient protective system capable of proactively identifying and mitigating risks. As a single-center retrospective analysis, the generalizability of these findings is limited. Moreover, potential risk factors such as adherence to standardized operating procedures and workload metrics were not quantitatively assessed, which may have resulted in the omission of key contributing variables. Future research should integrate multicenter data, incorporate richer process variables, and employ multivariable analytical approaches with long-term follow-up to advance occupational safety from empirical management toward precision prevention.

## Data Availability

The raw data supporting the conclusions of this article will be made available by the authors, without undue reservation.
